# Prevalence of MASLD and Fibrosis Risk in Turkish Adults with Cardiometabolic Risk Factors: A Nationwide Multicenter Study (DAHUDER MASLD Study)

**DOI:** 10.3390/jcm14197098

**Published:** 2025-10-08

**Authors:** Ali Kirik, Hilmi Erdem Sumbul, Nizameddin Koca, Türkan Paşalı Kilit, Sibel Demiral Sezer, Emine Binnetoglu, Eşref Araç, İhsan Solmaz, Hacer Şen, İbrahim Demirci, Bahri Abaylı, Hale Akan, Canan Akkuş, Berrin Aksakal, Gulali Aktas, Ömer Faruk Alakuş, Burçin Meryem Atak Tel, Ahmet Aydın, Sami Bahçebaşı, Orhan Balıkçı, Lale Saka Baraz, Bilgin Bahadır Başgöz, Muharrem Bayrak, Hilal Bektaş Uysal, Hatice Beyazal Polat, İfakat İrem Biçer, Rıfat Bozkuş, Fatih Coşkun, Cüneyt Çağatay, Feride Çağlar, Erkan Çakmak, Deniz Cekiç, Ülfet Değer, Ayse Kevser Demir, İsmail Demir, Oğuzhan Sıtkı Dizdar, Erkan Dulkadiroğlu, Nur Düzen Oflas, Betül Erişmiş, Ali Erol, Ayşegül Ertınmaz, Müzeyyen Eryılmaz, Emin Gemcioğlu, Ahmed Bilal Genç, Melis Gökgöz, Nevzat Gözel, Fatih İleri, Kubilay İşsever, Uğur Can İzlimek, Özge Kama Başcı, Aynur Kamburoğlu, Fatih Kamış, Sanem Kayhan, İsmail Kırlı, Yusuf Kimyon, Şeref Enes Kocaman, Kamil Konur, Özge Kurtkulağı, Celalettin Küçük, Mehmet Selim Mamiş, Hatice Metin, Necip Nas, Sibel Ocak Serin, Oktay Olmuşçelik, Alihan Oral, Muhammet Özbilen, Erkan Özdemir, Ensar Özmen, Hikmet Öztop, Huseyin Ali Ozturk, Osman Özüdoğru, Emel Sağlam, Hatice Özge Serin, Hasan Sözel, Cem Şahin, Melisa Şahin Tekin, Enes Seyda Şahiner, Ahmet Veli Şanibaş, Yasin Şahintürk, Hakan Şıvgın, Abdullah Tanrıkulu, Tuba Taslamacıoğlu Duman, Gokhan Tazegul, Elif Duygu Topan, Hasan Tunca, Seyit Uyar, Ece Ünal Çetin, Nazif Yalçın, Demet Yalçın Kehribar, Selçuk Yaylacı, Mehmet Serdar Yıldırım, Hasan Esat Yıldırım, Hüseyin Yıldız, Pınar Yıldız, Hasan Esat Yücel, Oğuzhan Zengin, Ali Zeynettin, Fatih Atik, Selin Müge Aslan, Mert Akyıldız, Nurcan Aslan, Sare Babacan Çelikel, Suat Baran Bakan, Merve Durmuş, Mert Karacay, Çağatay Koçyiğitoğlu, Gökçe Paşa, Selvinaz Sivri, Tutku Naz Şahin, Yağmur Sena Tosun, Zeynep Gizem Totik, Rabia Ulutaş, Tuğçe Nur Yazıcı, Emrah Yılmaz, Hamit Yıldız, Alper Sönmez, Teoman Doğru

**Affiliations:** 1 Department of Internal Medicine, Balikesir University Medical School, Balikesir 10145, Türkiye; alikirik87@hotmail.com (A.K.); hcrgrsy@hotmail.com (H.Ş.); ozgee.kama@gmail.com (Ö.K.B.); dr.atikfatih@gmail.com (F.A.); drslnasln@gmail.com (S.M.A.); 2 Department of Internal Medicine, Adana Health Practice and Research Center, University of Health Sciences, Adana 01230, Türkiye; erdemsumbul@gmail.com (H.E.S.); drozturkhuseyinali@gmail.com (H.A.O.); colt12377@gmail.com (M.A.); merveozdemiir01@gmail.com (M.D.); mertkaracay1@gmail.com (M.K.); cagatay.kocyigitoglu@gmail.com (Ç.K.); gokce.pasa@hotmail.com (G.P.); sivriselvinaz@gmail.com (S.S.); tutkunazcihan@gmail.com (T.N.Ş.); zgb_93@hotmail.com (Z.G.T.); 3 Department of Internal Medicine, Bursa City Hospital, Bursa 16250, Türkiye; nkoca@yahoo.com (N.K.); cnytcgty@gmail.com (C.Ç.); aertinmaz@yahoo.com (A.E.); fileri91@gmail.com (F.İ.); aynururhann@gmail.com (A.K.); hotickon@gmail.com (H.M.); nazifyalcin16@gmail.com (N.Y.); 4 Department of Internal Medicine, Kütahya Health Sciences University, Kütahya 43020, Türkiye; turkan.pasalikilit@ksbu.edu.tr; 5 Department of Internal Medicine, Izmir City Hospital, İzmir 35540, Türkiye; drdemiralsibel@yahoo.com (S.D.S.); drbalikci83@gmail.com (O.B.); 6 Department of Internal Medicine, Çorlu Vatan Hospital, Tekirdag 59850, Türkiye; edemirbas1@yahoo.com; 7 Department of Internal Medicine, Dicle University Medical School, Diyarbakir 21010, Türkiye; esrefarac@gmail.com; 8 Department of Internal Medicine, Diyarbakır Gazi Yasargil Training and Research Hospital, Diyarbakır 21010, Türkiye; ihsan2157@gmail.com (İ.S.); omerfaruk01@gmail.com (Ö.F.A.); bbbasgoz@gmail.com (B.B.B.); yildirs21@gmail.com (M.S.Y.); 9 Department of Endocrinology and Metabolism, Ankara Güven Hospital, Kavaklıdere, Ankara 06540, Türkiye; dr.idemirci@gmail.com (İ.D.); alpersonmez@yahoo.com (A.S.); 10 Department of Internal Medicine, Seyhan Hospital, Adana 01150, Türkiye; babayli@yahoo.com; 11 Department of Internal Medicine, Manisa City Hospital, Manisa 45040, Türkiye; drhale82@hotmail.com (H.A.); lalesaka6@gmail.com (L.S.B.); 12 Department of Internal Medicine, University of Health Sciences, Ankara Etlik City Training & Research Hospital, Ankara 06170, Türkiye; cananozkal@gmail.com (C.A.); rifatbozkus@gmail.com (R.B.); drferidecaglar@gmail.com (F.Ç.); egemcioglu@gmail.com (E.G.); drmelisgokgoz@gmail.com (M.G.); drkayhansanem@yahoo.com (S.K.); 13 Department of Internal Medicine, Umraniye Training and Research Hospital, Istanbul 34764, Türkiye; drberrincamur@gmail.com (B.A.); rdsibelocak@gmail.com (S.O.S.); 14 Department of Internal Medicine, Bolu Abant Izzet Baysal University Hospital, Bolu 14280, Türkiye; draliaktas@yahoo.com (G.A.); burcinatak@hotmail.com (B.M.A.T.); doktortuuba@gmail.com (T.T.D.); 15 Department of Internal Medicine, Faculty of Medicine, Istanbul Medipol University, Istanbul 34214, Türkiye; uzm.dr.ahmetaydin@gmail.com (A.A.); oolmuscelik@medipol.edu.tr (O.O.); 16 Department of Internal Medicine, Kayseri City Hospital, Kayseri 38080, Türkiye; doktorsami@yahoo.com (S.B.); iremkrtkn@gmail.com (İ.İ.B.); oguzhansitki.dizdar@sbu.edu.tr (O.S.D.); 17 Department of Internal Medicine, Erzurum Faculty of Medicine, Health Sciences University, Erzurum 25240, Türkiye; muharrem.bayrak@sbu.edu.tr; 18 Department of Internal Medicine, Faculty of Medicine, Aydın Adnan Menderes University, Aydın 09100, Türkiye; hilalbektasuysal@yahoo.com (H.B.U.); edtopan@adu.edu.tr (E.D.T.); 19 Department of Internal Medicine, Faculty of Medicine, Recep Tayyip Erdogan University, Rize 53020, Türkiye; drpolat53@hotmail.com; 20 Department of Internal Medicine, University of Health Sciences, Bursa Yüksek İhtisas Training and Research Hospital, Bursa 16310, Türkiye; fcooshkun@gmail.com (F.C.); alierol625@gmail.com (A.E.); eneskocamann@gmail.com (Ş.E.K.); hasanesatyldrm@gmail.com (H.E.Y.); 21 Department of Internal Medicine, Faculty of Medicine, Firat University, Elazığ 23119, Türkiye; drerkan_23@hotmail.com (E.Ç.); drngozel@hotmail.com (N.G.); bbcnsare44@gmail.com (S.B.Ç.); 22 Department of Internal Medicine, Faculty of Medicine, Sakarya University, Sakarya 54290, Türkiye; decekic@gmail.com (D.C.); ahmedbgenc@gmail.com (A.B.G.); selcukyaylaci@sakarya.edu.tr (S.Y.); 23 Department of Internal Medicine, Marmara University Pendik Training and Research Hospital, Istanbul 34899, Türkiye; ulfet.ursavas@gmail.com (Ü.D.); yusuf.kimyon@gmail.com (Y.K.); hozgeserin@gmail.com (H.Ö.S.); drgtazegul@gmail.com (G.T.); 24 Department of Internal Medicine, Samsun University Medical School, Samsun 55080, Türkiye; ayse.demir@samsun.edu.tr (A.K.D.); ahmetveli19@hotmail.com (A.V.Ş.); 25 Department of Internal Medicine, Bozyaka Training and Research Hospital, İzmir 35170, Türkiye; drismaildemir22@gmail.com (İ.D.); ataz6@hotmail.com (A.Z.); 26 Department of Internal Medicine, Ahi Evran University Medical School, Kirsehir 40200, Türkiye; erkan_dulkadir@hotmail.com (E.D.); drh.esat@hotmail.com (H.E.Y.); 27 Department of Internal Medicine, Faculty of Medicine, Van Yüzüncü Yıl University, Van 65090, Türkiye; dr.nurdzn@hotmail.com.tr; 28 Department of Internal Medicine, Ankara Bilkent City Hospital, Ankara 06800, Türkiye; drbetulerismis@gmail.com (B.E.); dr.enessahiner@gmail.com (E.S.Ş.); oguzhanzengin91@gmail.com (O.Z.); drnurcanaslan@gmail.com (N.A.); ygmrsenat@gmail.com (Y.S.T.); 29 Department of Internal Medicine, University of Health Sciences, Fatih Sultan Mehmet Education and Research Hospital, Istanbul 34752, Türkiye; drmuzeyyeneryilmaz@hotmail.com (M.E.); baranpirbari@gmail.com (S.B.B.); 30 Department of Internal Medicine, Faculty of Medicine, Giresun University, Giresun 28200, Türkiye; kubilayissever@gmail.com; 31 Department of Internal Medicine, 5 Ocak State Hospital, Adana 01210, Türkiye; ucizlimek@gmail.com; 32 Department of Internal Medicine, Çanakkale Onsekiz Mart University School of Medicine, Canakkale 17020, Türkiye; fatihkamis@hotmail.com (F.K.); ozgekurtkulagi@gmail.com (Ö.K.); eceunalcetin@gmail.com (E.Ü.Ç.); 33 Department of Internal Medicine, Faculty of Medicine, Muğla Sıtkı Koçman University, Muğla 48000, Türkiye; ismailkirli@mu.edu.tr (İ.K.); cemsahin@mu.edu.tr (C.Ş.); dr.hasan.tunca@gmail.com (H.T.); 34 Biruni University Vocational School, Istanbul 34020, Türkiye; celalettinkucuk@yahoo.com; 35 Department of Internal Medicine, Siirt University Medical School, Siirt 56100, Türkiye; dr.mehmetselim@outlook.com (M.S.M.); necipnas@gmail.com (N.N.); drabdt21@gmail.com (A.T.); 36 Department of Internal Medicine, Faculty of Medicine, Biruni University, Istanbul 34295, Türkiye; dr.alihanoral@gmail.com; 37 Department of Internal Medicine, Ordu University Training and Research Hospital, Ordu 52200, Türkiye; drozbilen@gmail.com (M.Ö.); tugcenuryazicii@gmail.com (T.N.Y.); 38 Department of Internal Medicine, Malatya Training and Research Hospital, Malatya 44300, Türkiye; erkanozdemir1989@gmail.com (E.Ö.); drhyildiz@yahoo.com (H.Y.); 39 Department of Internal Medicine, Kocaeli City Hospital, Kocaeli 41100, Türkiye; ensarozmen@hotmail.com; 40 Department of Internal Medicine, Faculty of Medicine, Bursa Uludag University, Bursa 16059, Türkiye; hikmetoztop@gmail.com; 41 Department of Internal Medicine, EBYU Medical School, Erzincan 24100, Türkiye; osmanozudogru2@gmail.com (O.Ö.); dr_emrahyilmaz@hotmail.com (E.Y.); 42 Department of Internal Medicine, University of Health Sciences, Bagcilar Training and Research Hospital, Istanbul 34200, Türkiye; dr.emelsaglam@hotmail.com; 43 Department of Internal Medicine, Akdeniz University Medical School, Antalya 07100, Türkiye; hasansozel@akdeniz.edu.tr; 44 Department of Internal Medicine, Faculty of Medicine, Eskisehir Osmangazi University, Eskisehir 26040, Türkiye; melisasahin@gmail.com (M.Ş.T.); pinaresogu@gmail.com (P.Y.); 45 Department of Internal Medicine, Antalya Training and Research Hospital, Antalya 07100, Türkiye; drsahinturk@yahoo.com (Y.Ş.); seyituyar79@hotmail.com (S.U.); 46 Department of Internal Medicine, Faculty of Medicine, Gaziosmanpasa University, Tokat 60100, Türkiye; sivginhakan@gmail.com; 47 Department of Internal Medicine, Faculty of Medicine, Dokuz Eylul University, İzmir 35330, Türkiye; kehribardemet@gmail.com (D.Y.K.); rabia.ulutas@deu.edu.tr (R.U.); 48 Department of Internal Medicine, Gaziantep University Medical School, Gaziantep 27850, Türkiye; drhyildiz@hotmail.com; 49 Department of Gastroenterology, Balikesir University Medical School, Balikesir 10145, Türkiye; teomandogru@yahoo.com

**Keywords:** MASLD, obesity, hypertension, type 2 diabetes mellitus, liver fibrosis, FIB-4 score

## Abstract

**Introduction**: Metabolic dysfunction-associated steatotic liver disease (MASLD) prevalence data in Türkiye is limited. We aimed to determine the nationwide prevalence of MASLD and advanced hepatic fibrosis risk in subjects with cardiometabolic risk factors (CMRF). Despite recent international consensus redefining fatty liver disease terminology, no nationwide MASLD study has been reported in Türkiye. **Methods**: This cross-sectional study included 14,371 adults with ≥1 CMRF from 44 centers across 31 cities. MASLD was diagnosed using liver ultrasonography plus cardiometabolic criteria. Advanced fibrosis risk was assessed by fibrosis-4 (FIB-4) score (≥1.3 for ≤65 years; ≥2.0 for >65 years). Logistic regression was used to identify independent predictors of high FIB-4. **Results**: A total of 61.4% of participants were women, the mean age was 51.3 ± 14.4 years, and the mean BMI was 31.4 ± 6.0 kg/m^2^. MASLD prevalence was 75.7% (n = 10,873), rising with the number of CMRFs (56.5% with one factor vs. 83.4% with all). The prevalence of high FIB-4 scores was 12.0% overall, being lower in MASLD patients than non-MASLD patients (11.2% vs. 14.4%, *p* < 0.001). FIB-4 scores decreased with increasing BMI (28.1% underweight vs. 8.7% class III obesity). Male sex, T2DM, and hypertension independently predicted high FIB-4 scores, while smoking, higher BMI, and MASLD were inversely associated. **Conclusions**: Three-quarters of Turkish adults with CMRF have MASLD. Standard FIB-4 thresholds may underestimate fibrosis risk in obese and smoking populations, underscoring the need for adjusted cut-offs or alternative tools. This study is the first to provide nationwide MASLD prevalence data in Türkiye.

## 1. Introduction

Metabolic dysfunction-associated steatotic liver disease (MASLD), formerly known as nonalcoholic fatty liver disease (NAFLD) and metabolic-associated fatty liver disease (MAFLD), is the most common chronic liver disease and is estimated to affect 30% of the adult population worldwide [1]. The presence of MASLD is closely associated with type 2 diabetes mellitus (T2DM), obesity, and other cardiometabolic risk factors (CMRFs) [2,3]. MASLD has rising prevalence and incidence in parallel with the increasing burden of obesity, T2DM, and metabolic syndrome (MetS) [4].

Liver fibrosis is the key determinant of liver-related mortality, particularly at the advanced fibrosis stage [5]. Consequently, the early diagnosis of advanced liver fibrosis is critical to improving the overall prognosis of chronic liver disease [6]. MASLD represents a rapidly increasing cause of liver cirrhosis and the fastest-growing etiology of hepatocellular carcinoma (HCC) [7]. The European Association for the Study of the Liver (EASL) recommends screening for liver fibrosis and steatotic liver disease in subjects with CMRF based on the following factors: excessive alcohol consumption, MetS, obesity, and T2DM [3]. The screening strategy relies on non-invasive tools [such as fibrosis-4 (FIB-4), the APRI, and NAFLD Fibrosis Score] and other methods [such as vibration-controlled transient elastography (VCTE), Enhanced Liver Fibrosis test, Fibrometer, and Fibrosure] [8].

The FIB-4 score is a simple test with no additional cost, based on aspartate aminotransferase (AST), alanine transaminase (ALT), platelets, and age [9]. If the FIB-4 score suggests advanced fibrosis, EASL recommends liver stiffness measurement to be performed using VCTE [3]. High FIB-4 scores are associated with an increased incidence of severe liver disease and liver-related outcomes in population-based studies [10]. Therefore, FIB-4 is recommended as a first-line assessment for fibrosis in subjects with CMRF, helping to identify those who require referral to a specialist liver clinic [11].

In recent years, international expert panels have redefined the terminology of fatty liver disease. A multisociety Delphi consensus, led by Rinella et al. [12], achieved greater than 75% agreement on adopting the MASLD term, underscoring the central role of metabolic risk factors in disease pathogenesis. Furthermore, Younossi et al. [13] emphasized in a global consensus statement that harmonizing MASLD/MASH definitions is essential for research comparability and clinical implementation. The 2023 EASL–EASD–EASO guidelines also highlight practical screening algorithms, risk stratification, and the limitations of currently used non-invasive tests [3]. In parallel, the Asia-Pacific Association for the Study of the Liver (APASL) issued 2025 recommendations tailored to regional epidemiology, again underscoring the need for context-specific strategies [14].

Beyond traditional non-invasive scores, recent advances in artificial intelligence (AI) and machine learning have been proposed for more accurate detection of steatosis and fibrosis. AI-driven models that combine anthropometric, biochemical, and imaging data have demonstrated improved diagnostic accuracy; however, external validation in large, diverse cohorts remains limited. The EASL Task Force on AI recently stressed the importance of validation, interpretability, and integration into clinical practice [15,16].

There is scarce data regarding the prevalence of MASLD in Türkiye. In several multicenter studies, the prevalence of NAFLD/MAFLD has been reported to exceed 30% [17,18]. However, there are no population-based studies that investigate the prevalence of MASLD and advanced hepatic fibrosis in Türkiye. Therefore, we aimed to examine (i) the prevalence of MASLD in a nationwide cohort of adult subjects with CMRF, (ii) assess hepatic fibrosis risk in subjects with MASLD, and (iii) determine the significant independent associates of MASLD and advanced fibrosis.

## 2. Materials and Methods

### 2.1. Study Design and Setting

This nationwide, multicenter, cross-sectional study was conducted between January and June 2024 in 44 internal medicine outpatient clinics across 31 cities of Türkiye. Clinical centers were selected to represent all 12 Nomenclature of Territorial Units for Statistics (NUTS-1) regions, thereby ensuring geographic and demographic diversity. Each center was instructed to consecutively recruit eligible participants during the study period. The study protocol was approved by the Local Ethics Committee of Balıkesir University (approval number: 2024/72; approval date: 21 May 2024) and was conducted in accordance with the principles of the Declaration of Helsinki. All participants provided written informed consent prior to enrollment.

### 2.2. Study Population

The study population consisted of adults with at least one cardiometabolic risk factor who presented to internal medicine outpatient clinics. Exclusion criteria were pregnancy, acute or chronic inflammatory diseases, active malignancy, prior bariatric surgery, and history of chronic liver disease due to secondary causes (including viral hepatitis, autoimmune liver diseases, Wilson’s disease, hemochromatosis, and significant alcohol consumption defined as >20 g/day for women and >30 g/day for men). Patients were enrolled consecutively during routine outpatient visits. A total of 14,884 individuals were initially screened; after excluding those with missing data (n = 373) or no risk factors (n = 140), 14,371 participants were included in the final analysis.

### 2.3. Data Collection and Anthropometric Measurements

A standardized case report form was used across all centers. Demographic data, medical history, and lifestyle habits (including smoking, alcohol use, and physical activity) were recorded by trained physicians. Physical activity was recorded through self-report. Participants were asked whether they engaged in at least 30 min of exercise per day. Body weight was measured to the nearest 0.1 kg using calibrated digital scales, with participants wearing light clothing without shoes. Height was measured with a stadiometer to the nearest 0.5 cm. Body mass index (BMI) was calculated as weight (in kilograms) divided by height (in meters squared). Waist circumference (WC) was measured using a flexible tape midway between the lower rib margin and the iliac crest at the end of normal expiration. Blood pressure (BP) was recorded three times in the seated position after a 5 min rest period using validated automated sphygmomanometers; the average of the three readings was used in analyses.

### 2.4. Laboratory Measurements

After an overnight fast of at least 8 h, venous blood samples were obtained. Laboratory analyses were performed in local hospital laboratories accredited by the Turkish Ministry of Health using standardized, quality-controlled equipment. Measurements included fasting plasma glucose (FPG), creatinine, aspartate aminotransferase (AST), alanine aminotransferase (ALT), total cholesterol (TC), triglycerides (TG), and high-density lipoprotein cholesterol (HDL-C). Low-density lipoprotein cholesterol (LDL-C) was calculated using the Friedewald formula [LDL-C = TC − (HDL-C + TG/5)] [18]. Platelet counts were determined using automated hematology analyzers.

### 2.5. Definition of Cardiometabolic Risk Factors and Metabolic Disorders

MASLD was diagnosed when hepatic steatosis on ultrasonography was accompanied by ≥1 CMRF according to EASL-EASD-EASO 2023 criteria [3].

Obesity: BMI ≥ 25 kg/m^2^ or WC ≥ 94 cm (men) or ≥80 cm (women).

Diabetes: FPG ≥ 126 mg/dL, 2 h post-load glucose ≥ 200 mg/dL, HbA1c ≥ 6.5%, or use of antidiabetic medication [19].

Hypertension: Office BP ≥ 140/90 mmHg, home BP ≥ 135/85 mmHg, or antihypertensive treatment [20].

Dyslipidemia: TG ≥ 150 mg/dL and/or LDL-C ≥ 100 mg/dL and/or HDL-C < 40 mg/dL in men or <50 mg/dL in women or lipid-lowering therapy [21].

Metabolic Syndrome: Defined according to NCEP ATP III criteria as the presence of ≥3 components [22].

Cardiovascular disease was defined as a documented history of angina, myocardial infarction, heart failure, or peripheral artery disease [23].

### 2.6. Hepatic Steatosis and Fibrosis Assessment

Hepatic steatosis was assessed by conventional abdominal ultrasonography performed by radiologists or experienced internists who were blinded to laboratory data. Steatosis was defined as increased echogenicity of the liver parenchyma relative to the renal cortex. Cases with inconclusive findings were excluded.

Fibrosis-4 (FIB-4) index was calculated using the following formula:

$$FIB-4=\frac{Age\left(years\right)\times AST(\frac{U}{L})}{Platelets\left(\frac{10^{9}}{L}\right)\times \sqrt{ALT(U/L)}}$$

High FIB-4 scores were defined as ≥1.3 in participants ≤65 years and ≥2.0 in those >65 years [3].

In addition to the FIB-4 index, we also calculated the AST-to-Platelet Ratio Index (APRI) as part of a sensitivity analysis for fibrosis risk. The APRI was computed using the following formula: [(AST/upper limit of normal [40 U/L]) × 100/platelet count (10^9^/L)]. A cut-off value of ≥0.7 was considered indicative of advanced fibrosis and ≥1.0 as significant fibrosis. Calculation of the NAFLD fibrosis score (NFS) was not possible due to the absence of serum albumin data in our dataset. However, the use of the APRI alongside FIB-4 enabled us to compare different non-invasive fibrosis scores within the same population.

### 2.7. Statistical Analysis

Statistical analyses were performed using SPSS version 25.0 (SPSS Inc., Chicago, IL, USA). Continuous variables were expressed as mean ± standard deviation (SD) or median (interquartile range, IQR) as appropriate. Categorical variables were summarized as counts (n) and percentages (%). Normality was tested with the Kolmogorov–Smirnov test. Between-group comparisons were made using Student’s *t*-test or the Mann–Whitney U test for continuous variables and the Chi-square test for categorical variables. Logistic regression models were built to identify independent determinants of high FIB-4 scores. Variables with *p* < 0.10 in univariate analyses and clinically relevant covariates were included in multivariable models. Missing data were handled by listwise deletion; cases with missing values for key variables were excluded from the relevant analyses without imputation. Statistical significance was defined as two-tailed *p* < 0.05.

## 3. Results

### 3.1. The Characteristics of Subjects According to the Diagnosis of MASLD

**Figure 1:**
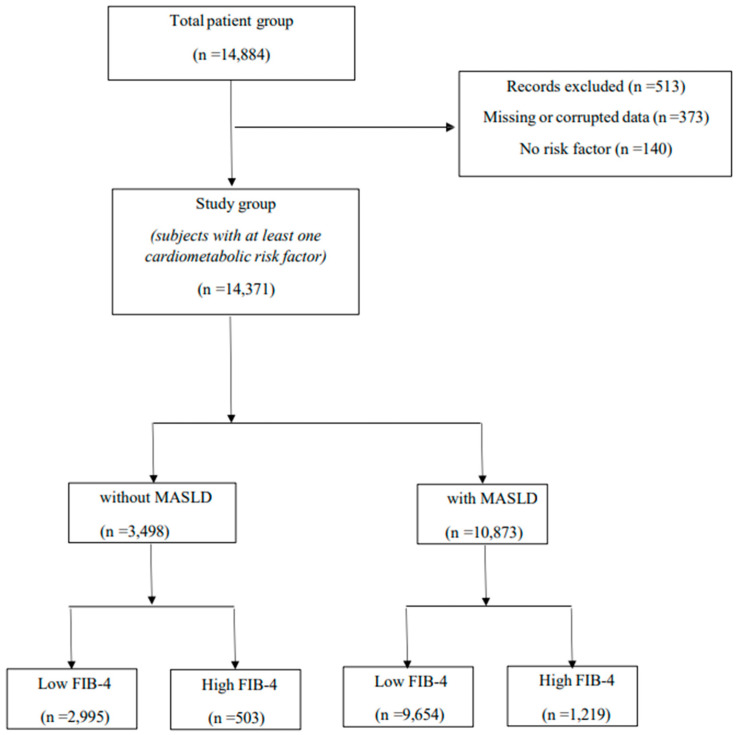
Flowchart of study population selection, exclusion criteria, and final stratification into MASLD-positive and MASLD-negative groups based on FIB-4 score categories.

In this clinical study, 513 of 14,884 patients [missing or corrupted data (n = 373), no risk factor (n = 140)] whose data were collected were excluded from the study (Figure 1). A total of 14,371 subjects (61.4% women) were enrolled in this study. Among the study participants, the prevalence of patients with MASLD was 75.7% (n = 10,873). The demographic, clinical, and laboratory parameters of subjects with and without MASLD are presented in Table 1.

Subjects with and without MASLD were of similar ages (*p* = 0.442). BMI levels were significantly higher in subjects with MASLD compared to those without MASLD (*p* < 0.001). The rates of obesity, T2DM, hypertension, dyslipidemia, and MetS were significantly higher in subjects with MASLD than those without MASLD (for all, *p* < 0.001). The mean FIB-4 levels were significantly lower in subjects with MASLD (0.96 ± 1.6) than those without MASLD (1.1 ± 1.1) (*p* = 0.001). In addition, the rate of high FIB-4 scores was significantly lower in subjects with MASLD [n = 1219 (11.2%)] than those without MASLD [n = 503 (14.4%)] (*p* < 0.001).

In the subgroup analysis performed according to comorbid conditions, the prevalence of MASLD in patients with dyslipidemia was 80.9% (compared to 71.4% in those without dyslipidemia), 79.6% in patients with T2DM (compared to 72.7% in those without T2DM), and 78.1% in patients with hypertension (compared to 73.9% in those without hypertension) and was found at the highest rate (for all, *p* < 0.001).

### 3.2. The Characteristics of Subjects According to the FIB-4 Score

The demographic, clinical, and laboratory parameters of participants, categorized by the FIB-4 score, are presented in Table 2. Subjects with low FIB-4 scores had higher BMI levels compared to those with high FIB-4 scores (31.6 ± 6.0 kg/m^2^ and 30.4 ± 5.8 kg/m^2^, respectively) (*p* < 0.001). Subjects with high FIB-4 scores had higher rates of T2DM, HT, and MetS (*p* < 0.001 for all).

### 3.3. The Prevalence of MASLD and High FIB-4 Scores According to CMRFs

**Figure 2:**
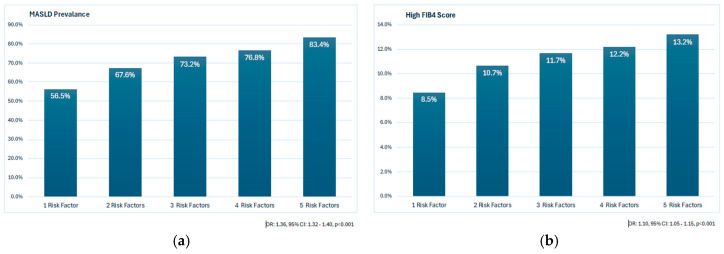
Association between the number of cardiometabolic risk factors and (**a**) MASLD prevalence and (**b**) the proportion of patients with high FIB-4 scores.

The prevalence of MASLD progressively increased with the number of CMRFs, ranging from 56.5% in participants with one factor to 83.4% in those with all five factors (Figure 2a). In subgroup analyses, MASLD prevalence was particularly high among participants with both type 2 diabetes and hypertension (80.9%) compared with 77.6% in those with diabetes alone, 73.6% in those with hypertension alone, and 72.3% in those with neither condition (Table 3). Similarly, the prevalence of high FIB-4 scores increased modestly with the number of CMRFs, from 8.5% with one factor to 13.2% with five factors (Figure 2b).

### 3.4. The Prevalence of MASLD and High FIB-4 Scores According to BMI

**Figure 3:**
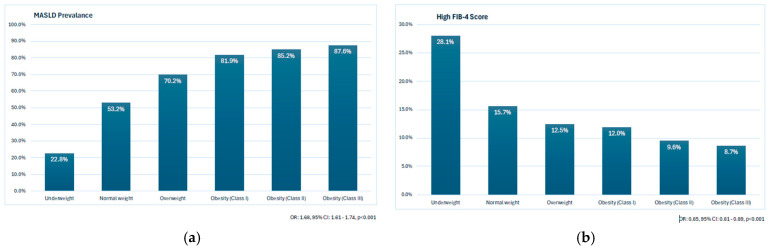
Relationship between body mass index (BMI) categories and (**a**) MASLD prevalence and (**b**) prevalence of high FIB-4 scores.

The prevalence of MASLD increased in tandem with rising BMI levels. MASLD prevalence was 22.8% in underweight patients, 53.2% in normal-weight patients, 70.2% in patients with overweight, 81.9% in patients with obesity class 1, 85.2% in patients with obesity class 2, and 87.6% in patients with obesity class 3 (Figure 3a). Conversely, the prevalence of high FIB-4 scores decreased along with increasing BMI. The prevalence of high FIB-4 scores was 28.1% in underweight patients, 15.7% in normal-weight patients, 12.5% in patients with overweight, 12.0% in patients with obesity class 1, 9.6% in patients with obesity class 2, and 8.7% in patients with obesity class 3 (Figure 3b).

### 3.5. The Independent Determinants of High FIB-4 Scores

**Figure 4:**
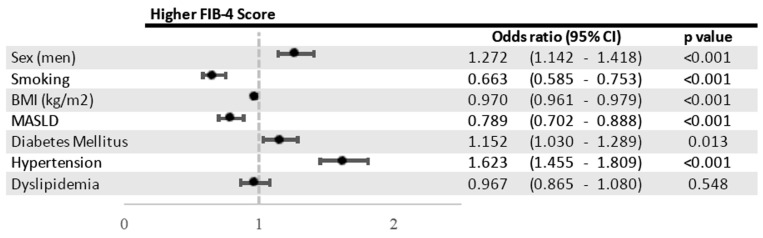
Forest plot of multivariable logistic regression analysis showing independent factors associated with higher FIB-4 scores.

In the multivariable model, the male sex (OR: 1.272, 95% CI: 1.142–1.418), BMI (OR: 0.970, 95% CI: 0.961–0.979), smoking (OR: 0.663, 95% CI: 0.585–0.753), MASLD (OR: 0.789, 95% CI: 0.702–0.888), hypertension (OR: 1.623, 95% CI: 1.455–1.809) (for all *p* < 0.001), and T2DM (OR: 1.152, 95% CI: 1.030–1.289) (*p* = 0.013) were significant independent associates of liver fibrosis (Figure 4).

### 3.6. Sensitivity Analysis

In a sensitivity analysis using the AST-to-Platelet Ratio Index (APRI), only 2.6% of participants met the advanced fibrosis threshold (≥0.7), and 1.4% met the higher threshold (≥1.0), which was substantially lower than the prevalence estimated using age-adjusted FIB-4 scores (12.4%). (Table 4).

## 4. Discussion

This nationwide, multicenter, cross-sectional study shows a high prevalence of MASLD in Türkiye. Three in four people living with at least one CMRF have MASLD. The results also show an increasing prevalence of MASLD and high FIB-4 scores along with a rising number of CMRFs. However, the prevalence of high FIB-4 scores decreases as BMI levels increase. The independent positive associates of high FIB-4 levels were the male sex, T2DM, and HT, while smoking, higher BMI, and MASLD were significant negative associates.

MASLD is currently defined as hepatic steatosis in individuals with at least one CMRF [3]. This study represents the first national MASLD prevalence study conducted worldwide according to this new definition. Our literature review revealed that only one editor’s letter has been published so far on MASLD prevalence. This letter, based on NHANES 2017–2020 participant data from the United States, reported a MASLD prevalence of 83.7% among individuals with all five CMRFs [24]. This rate was found to be 83.4% in our study, demonstrating that our findings on MASLD prevalence are very similar to those from the US population. Although no other published data on MASLD prevalence exists, it has been reported that epidemiological data based on the former definition of NAFLD largely resemble MASLD data, with only 5% of patients diagnosed with NAFLD failing to meet MASLD criteria [1]. Our data demonstrate that MASLD prevalence in Turkish adults is 75.7%. This figure indicates that MASLD prevalence in our country is higher than the NAFLD prevalence reported in previously published studies with limited national representativeness [17,25]. Epidemiological evidence shows that the Middle East and North Africa (MENA) region has the highest global prevalence of MASLD, with Türkiye having the second highest prevalence in this region after Egypt [26]. The increasing prevalence of obesity and T2DM in Türkiye and the surrounding region plays a major role in the increasing prevalence of MASLD [27,28].

The present study and previous investigations have reaffirmed that MASLD occurs in most adults with CMRF. Regarding the high prevalence of MASLD in subjects with CMRF, it is reasonable to prioritize hepatic fibrosis risk screening over MASLD screening in this population. Our subgroup analysis demonstrated that MASLD prevalence was highest in individuals with coexisting diabetes and hypertension (80.9%). This synergistic effect of clustered metabolic comorbidities has also been emphasized in previous studies. Lonardo et al. reported that the coexistence of hypertension and diabetes accelerates the progression of NAFLD and increases the risk of cardiovascular disease [29]. Similarly, Fu et al. found that hypertension was independently associated with both the presence and severity of NAFLD [30]. Byrne and colleagues highlighted the cumulative impact of metabolic syndrome components, particularly diabetes and hypertension, on hepatic and cardiovascular outcomes [31]. Collectively, these findings support the need for intensified screening strategies in high-risk groups, especially patients with multiple metabolic disorders. Liver fibrosis is the main determinant of long-term liver-related outcomes in chronic liver diseases. Early diagnosis of fibrosis and subsequent appropriate management can prevent progression to cirrhosis and its complications [32]. Research in large cohorts has shown that hepatic fibrosis as a result of MASLD is associated with major adverse liver outcomes and all-cause mortality [33]. Several non-invasive markers of fibrosis have emerged as alternatives to staging fibrosis through liver biopsy. The FIB-4 score is the most convenient and common non-invasive tool used to determine the presence of advanced liver fibrosis in MASLD. Because a high FIB-4 score strongly predicts severe liver outcomes regardless of known SLD status, major guidelines recommend using FIB-4 as the initial non-invasive tool for risk stratification in subjects with MASLD or CMRF [3,34,35].

Beyond FIB-4, we also assessed the APRI to explore consistency across non-invasive scores. Interestingly, the APRI identified only 2–3% of participants as high risk, in contrast to the 12% identified by FIB-4. This striking discrepancy is consistent with prior reports that the APRI underestimates fibrosis in metabolic populations [10]. These findings highlight the need for population-specific validation of fibrosis scores and illustrate the limitations of applying uniform cut-offs across diverse clinical groups.

This is the first study to search for the national prevalence of advanced hepatic fibrosis risk in Turkish patients with MASLD. In patients with at least one CMRF, the overall prevalence of high FIB-4 scores was 12.0%. Moreover, the risk of developing hepatic fibrosis increased as the number of CMRFs increased. It is worth noting that our study population consisted exclusively of adults with at least one cardiometabolic risk factor; therefore, the findings cannot be generalized to the entire Turkish adult population. Instead, our results specifically reflect the burden of MASLD and the risk of fibrosis among individuals at high risk. However, contrary to expectations, our findings demonstrated lower rates of elevated FIB-4 scores in individuals with MASLD, those with high BMI, and current smokers. Obesity is closely associated with an increased risk of progression to moderate-to-advanced liver fibrosis, both in the general population and in patients with MASLD [36]. Despite this consistently reported association, our findings revealed a paradoxical inverse relationship between FIB-4 scores and obesity, with both FIB-4 levels and the prevalence of elevated scores decreasing significantly as BMI increased. Obesity, a chronic, low-grade systemic inflammatory condition, is associated with increased circulating platelet levels [37]. Since FIB-4 includes platelet count in the denominator, higher platelet counts in subjects with obesity result in lower FIB-4 scores. Likewise, the FIB-4 score was significantly lower in subjects who were smokers than in non-smokers, and smoking was inversely and significantly associated with the FIB-4 score in the present study. Cigarette smoking is a well-recognized driver of fibrosis progression in various liver pathologies, including MASLD [38]. This paradoxically lower FIB-4 score observed in smokers can again be attributed to their elevated platelet counts [39]. In addition, conventional FIB-4 cut-offs were not developed in populations with high obesity prevalence, which may partly explain the underestimation of fibrosis risk in these groups. While ultrasonography has limited sensitivity for detecting mild steatosis, it is not sufficient to attribute the paradox solely to imaging limitations. The overall data suggest that standard FIB-4 thresholds may underestimate advanced fibrosis risk in obese and smoking populations, underscoring the need for BMI-adjusted cut-offs or alternative non-invasive tools rather than rendering current thresholds entirely inappropriate [40].

Interestingly, another unexpected finding of the present study was the lower FIB-4 scores in subjects with MASLD compared to those without MASLD. In our opinion, the most important reason for this is the higher prevalence of obesity and smoking in MASLD cases. Since FIB-4 scores decrease with increasing BMI and smoking, it is reasonable to find lower FIB-4 scores in MASLD cases. Moreover, liver ultrasonography has some limitations in evaluating liver fat, including high inter-observer variability, low sensitivity for detecting mild steatosis (liver fat content < 20%), and lower accuracy in patients with liver fibrosis [41]. While these limitations may have contributed to misclassification, it would be inaccurate to conclude that ultrasonography is not a reliable method. Instead, our results emphasize the need for complementary diagnostic approaches, including advanced imaging or non-invasive biomarkers, particularly in populations with high cardiometabolic risk.

Several studies have non-invasively evaluated the risk of hepatic fibrosis in general populations; however, the results are inconsistent regarding sex differences [42]. MASLD is a sex-dimorphic disease, and its prevalence and severity are higher in men than in women during the reproductive age [43]. However, after menopause, MASLD occurs at a higher rate in women, suggesting that estrogen is protective. Several studies reported that postmenopausal women showed a significant association with an increased risk of diabetes, hypertriglyceridemia, and central obesity, which are important risk factors for MASLD [44]. In addition, it has been reported that once MASLD is established, women have a higher risk of advanced fibrosis than men, especially after 50 years of age [45,46]. In the present study, subjects with high FIB-4 scores were older than subjects with low FIB-4 scores. Moreover, a significant and negative association of the female sex with advanced fibrosis was observed. Although we did not evaluate menopause status in the present study, our findings provide further support that sex is an important factor regarding liver fibrosis.

Because all participants were recruited from internal medicine outpatient clinics, our findings represent a high-risk clinical sample rather than the general Turkish population. Therefore, the results should be interpreted in this context and not extrapolated to population-based prevalence estimates.

The close association between MASLD and clustered cardiometabolic risk factors observed in our cohort underscores the central role of lifestyle modification, including dietary changes, weight reduction, and regular exercise, as the cornerstone of management. Current EASL–EASD–EASO guidelines recommend targeted lifestyle interventions and cardiometabolic risk reduction as the first-line approach [3]. Recently, pharmacological options have also emerged: resmetirom received FDA approval in 2024 as the first therapy for metabolic dysfunction-associated steatohepatitis with fibrosis [47], and GLP-1 receptor agonists such as semaglutide [48] and dual agonists like tirzepatide [49] have shown efficacy in improving liver histology through weight loss and metabolic control. These advances highlight the therapeutic potential of addressing both hepatic and extra-hepatic risk factors. Our findings suggest that patients with comorbidities including diabetes, hypertension, and obesity represent a particularly high-risk subgroup that may derive the greatest benefit from such emerging therapies.

## 5. Conclusions

This nationwide multicenter study reveals that MASLD affects three-quarters of Turkish adults with CMRF, representing a substantial public health burden that parallels global trends in metabolic diseases. The results show a clear dose–response relationship between the number of CMRFs and MASLD prevalence, emphasizing the systemic nature of this condition. However, the paradoxically lower FIB-4 scores observed in patients with MASLD, obesity, and smoking history highlight critical limitations of current non-invasive fibrosis assessment tools in these high-risk populations. The inverse relationship between BMI and FIB-4 scores, primarily driven by elevated platelet counts in obesity, suggests that standard FIB-4 thresholds may systematically underestimate the risk of advanced fibrosis in vulnerable groups. These findings underscore the need for refined, BMI-adjusted FIB-4 cut-offs or alternative non-invasive tools validated specifically for obese and smoking populations. Importantly, our estimates reflect adults with cardiometabolic risk factors and should not be generalized to the entire Turkish population. Future studies incorporating advanced imaging, histological validation, and emerging pharmacotherapies are warranted to optimize MASLD detection and management strategies.

### 5.1. Strengths and Limitations

The strengths of this study include its prospective, multicenter nature and well-characterized participants from different regions of the country. The use of standardized diagnostic criteria enhances global comparability, while the systematic evaluation of dose–response relationships between CMRFs and MASLD provides valuable epidemiological insights. However, several limitations must be acknowledged. Exercise was assessed via self-reporting and dichotomized as ≥30 min per day versus none; however, frequency beyond daily reporting, duration over longer time frames, and exercise intensity were not captured, which may limit the interpretability of the results. Conventional ultrasonography has inherent limitations, including reduced sensitivity for mild steatosis and operator dependency. Although both FIB-4 and APRI were evaluated, these surrogate markers yielded divergent prevalence estimates, underlining their limited diagnostic reliability. Furthermore, the absence of elastography, ELF testing, or biopsy confirmation prevented validation of the fibrosis assessment, and serum albumin data were unavailable for computing the NAFLD fibrosis score. Another limitation is that only adults with at least one cardiometabolic risk factor were included in this study, and all participants were recruited from internal medicine outpatient clinics. Thus, our prevalence estimates are not representative of the general population but rather of a selected high-risk clinical subgroup.

### 5.2. Future Research Directions

Future studies should focus on validating BMI-adjusted or population-specific FIB-4 thresholds, integrating alternative biomarkers and advanced imaging methods, and exploring artificial intelligence-based diagnostic approaches. Longitudinal follow-up will also be crucial in determining the prognostic implications of MASLD and in designing tailored screening strategies for different cardiometabolic subgroups.

## Figures and Tables

**Table 1:** Demographic and metabolic characteristics of the study population.

Variables	Total Population n = 14,371	With MASLD n = 10,873 (75.7%)	Without MASLD n = 3498 (24.3%)	*p* Values
Demographic parameters				
Age (year) *	51.3 ± 14.4	51.4 ± 16.42	51.2 ± 13.6	0.442
Sex (women) **	8827 (61.4)	6503 (59.8)	2324 (66.4)	<0.001
BMI (kg/m^2^) *	31.4 ± 6.0	32.2 ± 10.0	29.1 ± 5.6	<0.001
Smoking **	3996 (27.8)	3090 (28.4)	906 (25.9)	0.004
Exercise **	2804 (19.5)	1902 (17.5)	902 (25.8)	<0.001
Comorbid diseases				
T2DM **	6181 (43.0)	4919 (45.2)	1262 (36.1)	<0.001
Hypertension **	6104 (42.5)	4765 (43.8)	1339 (38.3)	<0.001
Dyslipidemia **	6403 (44.6)	5183 (47.7)	1220 (34.9)	<0.001
MetS **	11,544 (80.3)	9062 (83.3)	2482 (71.0)	<0.001
Obesity **	7822 (54.4)	6544 (60.2)	1278 (36.5)	<0.001
Laboratory and markers				
Platelet (×103) *	276.0 ± 77.0	277.5 ± 77.8	270.1 ± 73.3	<0.001
AST (IU/L) *	24.2 ± 24.5	24.4 ± 22.6	23.6 ± 30.9	0.126
ALT (IU/L) *	28.7 ± 32.7	28.9 ± 30.8	27.5 ± 39.5	0.032
FIB-4 score *	0.99 ± 1.5	0.96 ± 1.6	1.1 ± 1.1	0.001
High FIB-4 score **	1722 (12.0)	1219 (11.2)	503 (14.4)	<0.001

Continuous variables are presented as * mean ± SD, and categorical data as ** n (%). MASLD, metabolic dysfunction-associated steatotic liver disease; BMI, body mass index; T2DM, type 2 diabetes mellitus; MetS, metabolic syndrome; AST, aspartate aminotransferase; ALT, alanine aminotransferase; FIB-4, fibrosis-4.

**Table 2:** Demographic and metabolic parameters of the study population according to FIB-4 status.

Variables	Total Population n = 14,371	Low FIB-4 Score n = 12,649 (88.0%)	High FIB-4 Score n = 1722 (12.0%)	*p* Values
Demographic parameters				
Age (year) *	51.3 ± 14.4	50.1 ± 14.4	60.1 ± 10.9	<0.001
Sex (women) **	8827 (61.4)	7834 (62.0)	984 (57.1)	<0.001
Smoking **	3996 (27.8)	3627 (28.7)	369 (21.4)	<0.001
Exercise **	2804 (19.5)	2527 (20.0)	277 (16.1)	<0.001
BMI (kg/m^2^) *	31.4 ± 6.0	31.6 ± 6.0	30.4 ± 5.8	<0.001
Comorbid diseases				
T2DM **	6181 (43.0)	5337 (42.2)	844 (49.0)	<0.001
Hypertension **	6104 (42.5)	5183 (41.0)	921 (53.5)	<0.001
Dyslipidemia **	6403 (44.6)	5598 (44.3)	805 (46.7)	0.051
MetS **	11,544 (80.3)	10,104 (79.9)	1440 (83.6)	<0.001
Obesity **	7822 (54.4)	6974 (55.1)	848 (49.2)	<0.001
MASLD **	10,873 (75.7)	9654 (76.3)	1219 (70.8)	<0.001
Laboratory and markers				
Platelet (×103) *	276.0 ± 77.0	286.8 ± 72.7	196.5 ± 59.7	<0.001
AST (IU/L) *	24.2 ± 24.5	21.5 ± 10.3	44.1 ± 61.3	<0.001
ALT (IU/L) *	28.7 ± 32.7	27.1 ± 24.5	39.9 ± 66.2	<0.001
FIB-4 score *	0.99 ± 1.5	0.79 ± 0.34	2.42 ± 3.82	<0.001

Continuous variables are presented as * mean ± SD, and categorical data as ** n (%). FIB-4, fibrosis-4; BMI, body mass index; T2DM, type 2 diabetes mellitus; MetS, metabolic syndrome; AST, aspartate aminotransferase; ALT, alanine aminotransferase; MASLD, metabolic dysfunction-associated steatotic liver disease.

**Table 3:** MASLD prevalence according to diabetes and hypertension status.

Group	Total (n)	MASLD (n)	Prevalence (%)
DM (+)/HT (+)	3730	3017	80.9%
DM (+)/HT (−)	2451	1902	77.6%
DM (−)/HT (+)	2374	1748	73.6%
DM (−)/HT (−)	5816	4206	72.3%

MASLD, metabolic dysfunction-associated steatotic liver disease; DM, diabetes mellitus; HT, hypertension.

**Table 4:** Comparison of non-invasive fibrosis scores in the study population.

Score	n	%
FIB-4 (age-adjusted)	1778	12.4
FIB-4 ≥ 2.67	330	2.3
APRI ≥ 0.7	375	2.6
APRI ≥ 1.0	207	1.4

FIB-4, fibrosis-4; APRI, AST-to-Platelet Ratio Index.

## Data Availability

The data presented in this study are available upon reasonable request from the corresponding author. The data are not publicly available due to privacy and ethical restrictions.

## References

[1] Younossi Z.M., Paik J.M., Stepanova M., Ong J., Alqahtani S., Henry L. (2024). Clinical profiles and mortality rates are similar for metabolic dysfunction-associated steatotic liver disease and non-alcoholic fatty liver disease. J. Hepatol..

[2] Kirik A., Dogru T., Yanik B., Sen H., Eroglu M., Baykan O., Bozyel E.A., Ergene A., Selcuk E., Tasci I. (2023). The relationship of circulating MOTS-c level with liver fibrosis and metabolic components in patients with metabolic dysfunction-associated fatty liver disease. Eur. Rev. Med. Pharmacol. Sci..

[3] European Association for the Study of the Liver (EASL), European Association for the Study of Diabetes (EASD), European Association for the Study of Obesity (EASO) (2024). EASL-EASD-EASO Clinical Practice Guidelines on the management of metabolic dysfunction-associated steatotic liver disease (MASLD). J. Hepatol..

[4] Oral A., Solmaz I., Koca N., Topaloglu U.S., Demir I., Dundar A., Kirik A., Basci O.K., Sen H., Binnetoglu E. (2025). Obesity-Related Disorders in Türkiye: A Multi Center, Retrospective, Cross-Sectional Analysis from the OBREDI-TR Study. J. Clin. Med..

[5] Ng C.H., Lim W.H., Lim G.E.H., Tan D.J.H., Syn N., Muthiah M.D., Huang D.Q., Loomba R. (2023). Mortality Outcomes by Fibrosis Stage in Nonalcoholic Fatty Liver Disease: A Systematic Review and Meta-analysis. Clin. Gastroenterol. Hepatol..

[6] Mignot V., Chirica C., Tron L., Borowik A., Borel A.L., Rostaing L., Bouillet L., Decaens T., Guergour D., Costentin C.E. (2024). Early screening for chronic liver disease: Impact of a FIB-4 first integrated care pathway to identify patients with significant fibrosis. Sci. Rep..

[7] Phoolchund A.G.S., Khakoo S.I. (2024). MASLD and the Development of HCC: Pathogenesis and Therapeutic Challenges. Cancers.

[8] Dietrich C.G., Rau M., Geier A. (2021). Screening for nonalcoholic fatty liver disease-when, who and how?. World J. Gastroenterol..

[9] Loomba R., Adams L.A. (2020). Advances in non-invasive assessment of hepatic fibrosis. Gut.

[10] Lee J., Vali Y., Boursier J., Spijker R., Anstee Q.M., Bossuyt P.M., Zafarmand M.H. (2021). Prognostic accuracy of FIB-4, NAFLD fibrosis score and APRI for NAFLD-related events: A systematic review. Liver Int..

[11] Şahintürk Y., Köker G., Koca N., Sümbül H.E., Demir İ., Keskin H., Yaylacı S., Solmaz İ., Açmaz B., Yıldız H. (2024). Metabolic Dysfunction-Associated Fatty Liver Disease and Fibrosis Status in Patients with Type 2 Diabetes Treated at Internal Medicine Clinics: Türkiye DAHUDER Awareness of Fatty Liver Disease (TR-DAFLD) Study. Turk. J. Gastroenterol..

[12] Rinella M.E., Lazarus J.V., Ratziu V., Francque S.M., Sanyal A.J., Kanwal F., Romero D., Abdelmalek M.F., Anstee Q.M., Arab J.P. (2024). A multisociety Delphi consensus statement on new fatty liver disease nomenclature. Ann. Hepatol..

[13] Younossi Z.M., Zelber-Sagi S., Lazarus J.V., Wong V.W.-S., Yilmaz Y., Duseja A., Eguchi Y., Castera L., Pessoa M.G., Oliveira C.P. (2025). Global Consensus Recommendations for Metabolic Dysfunction-Associated Steatotic Liver Disease and Steatohepatitis. Gastroenterology.

[14] Eslam M., Fan J.-G., Yu M.-L., Wong V.W.-S., Cua I.H., Liu C.-J., Tanwandee T., Gani R., Seto W.-K., Alam S. (2025). The Asian Pacific association for the study of the liver clinical practice guidelines for the diagnosis and management of metabolic dysfunction-associated fatty liver disease. Hepatol. Int..

[15] Clusmann J., Balaguer-Montero M., Bassegoda O., Schneider C.V., Seraphin T., Paintsil E., Luedde T., Lopez R.P., Calderaro J., Gilbert S. (2025). The barriers for uptake of artificial intelligence in hepatology and how to overcome them. J. Hepatol..

[16] Gao B., Duan W. (2025). The current status and future directions of artificial intelligence in the prediction, diagnosis, and treatment of liver diseases. Digit Health..

[17] Yilmaz Y., Yilmaz N., Ates F., Karakaya F., Gokcan H., Kaya E., Adali G., Kartal A.C., Sen I., Ahishali E. (2021). The Prevalence of Metabolic Associated Fatty Liver Disease in The Turkish Population: A Multicenter Study. Hepatol. Forum.

[18] Degertekin B., Tozun N., Demir F., Soylemez G., Parkan S., Gurtay E., Mutlu D., Toraman M., Seymenoglu T.H. (2021). The Changing Prevalence of Non-Alcoholic Fatty Liver Disease (NAFLD) in Turkey in the Last Decade. Turk. J. Gastroenterol..

[19] Friedewald W.T., Levy I.R., Fredrickson D.S. (1972). Estimation of the Concentration of Low-Density Lipoprotein Cholesterol in Plasma, Without Use of the Preparative Ultracentrifuge. Clin. Chem..

[20] (2025). American Diabetes Association Professional Practice Committee. 2. Diagnosis and Classification of Diabetes: Standards of Care in Diabetes-2025. Diabetes Care.

[21] McEvoy J.W., McCarthy C.P., Bruno R.M., Brouwers S., Canavan M.D., Ceconi C., Christodorescu R.M., Daskalopoulou S.S., Ferro C.J., Gerdts E. (2024). 2024 ESC Guidelines for the management of elevated blood pressure and hypertension. Eur. Heart J..

[22] Zeitouni M., Sabouret P., Kerneis M., Silvain J., Collet J.-P., Bruckert E., Montalescot G. (2021). 2019 ESC/EAS Guidelines for management of dyslipidaemia: Strengths and limitations. Eur. Heart J.—Cardiovasc. Pharmacother..

[23] Huang P.L. (2009). A comprehensive definition for metabolic syndrome. Dis. Model. Mech..

[24] Olvera Lopez E., Ballard B.D., Jan A. (2023). Cardiovascular Disease. StatPearls.

[25] Yang T., Yin J., Li J., Wang Q. (2024). The influence of different combinations of cardiometabolic risk factors on the prevalence of MASLD and risk of advanced fibrosis deserves attention. J. Hepatol..

[26] Sezgin O., Akpinar H., Ozer B., Toruner M., Bal K., Bor S. (2023). The Abdominal Ultrasonography Results of Cappadocia Cohort Study of Turkey Reveals High Prevalence of Fatty Liver. Turk. J. Gastroenterol..

[27] Younossi Z.M., Golabi P., Paik J., Owrangi S., Yilmaz Y., El-Kassas M., Alswat K., Alqahtani S.A. (2024). Prevalence of metabolic dysfunction-associated steatotic liver disease in the Middle East and North Africa. Liver Int..

[28] NCD Risk Factor Collaboration (NCD-RisC) (2024). Worldwide trends in diabetes prevalence and treatment from 1990 to 2022: A pooled analysis of 1108 population-representative studies with 141 million participants. Lancet.

[29] NCD Risk Factor Collaboration (NCD-RisC) (2024). Worldwide trends in underweight and obesity from 1990 to 2022: A pooled analysis of 3663 population-representative studies with 222 million children, adolescents, and adults. Lancet.

[30] Lonardo A., Nascimbeni F., Mantovani A., Targher G. (2018). Hypertension, diabetes, atherosclerosis and NASH: Cause or consequence?. J. Hepatol..

[31] Fu H., Yu H., Zhao Y., Chen J., Liu Z. (2023). Association between hypertension and the prevalence of liver steatosis and fibrosis. BMC Endocr. Disord..

[32] Byrne C.D., Targher G. (2022). Non-alcoholic fatty liver disease-related risk of cardiovascular disease and other cardiac complications. Diabetes, Obes. Metab..

[33] Somnay K., Wadgaonkar P., Sridhar N., Roshni P., Rao N., Wadgaonkar R. (2024). Liver Fibrosis Leading to Cirrhosis: Basic Mechanisms and Clinical Perspectives. Biomedicines.

[34] Ochoa-Allemant P., Hubbard R.A., Kaplan D.E., Serper M. (2025). Adverse Liver Outcomes, Cardiovascular Events, and Mortality in Steatotic Liver Disease. JAMA Intern. Med..

[35] Schreiner A.D., Moran W.P., Zhang J., Livingston S., Marsden J., Mauldin P.D., Koch D., Gebregziabher M. (2022). The Association of Fibrosis-4 Index Scores with Severe Liver Outcomes in Primary Care. J. Gen. Intern. Med..

[36] Rinella M.E., Neuschwander-Tetri B.A., Siddiqui M.S., Abdelmalek M.F., Caldwell S., Barb D., Kleiner D.E., Loomba R. (2023). AASLD Practice Guidance on the clinical assessment and management of nonalcoholic fatty liver disease. Hepatology.

[37] Julián M.T., Arteaga I., Torán-Monserrat P., Pera G., de Oca A.P.-M., Ruiz-Rojano I., Casademunt-Gras E., Chacón C., Alonso N. (2024). The Link between Abdominal Obesity Indices and the Progression of Liver Fibrosis: Insights from a Population-Based Study. Nutrients.

[38] Purdy J.C., Shatzel J.J. (2021). The hematologic consequences of obesity. Eur. J. Haematol..

[39] Akhavan Rezayat A., Dadgar Moghadam M., Ghasemi Nour M., Shirazinia M., Ghodsi H., Rouhbakhsh Zahmatkesh M.R., Tavakolizadeh Noghabi M., Hoseini B., Akhavan Rezayat K. (2018). Association between smoking and nonalcoholic fatty liver disease: A systematic review and meta-analysis. SAGE Open Med..

[40] Ghahremanfard F., Semnani V., Ghorbani R., Malek F., Behzadfar A., Zahmatkesh M. (2015). Effects of cigarette smoking on morphological features of platelets in healthy men. Saudi Med. J..

[41] Eren F., Kaya E., Yilmaz Y. (2022). Accuracy of Fibrosis-4 index and non-alcoholic fatty liver disease fibrosis scores in metabolic (dysfunction) associated fatty liver disease according to body mass index: Failure in the prediction of advanced fibrosis in lean and morbidly obese individuals. Eur. J. Gastroenterol. Hepatol..

[42] Bozic D., Podrug K., Mikolasevic I., Grgurevic I. (2022). Ultrasound Methods for the Assessment of Liver Steatosis: A Critical Appraisal. Diagnostics.

[43] Lonardo A., Nascimbeni F., Ballestri S., Fairweather D., Win S., Than T.A., Abdelmalek M.F., Suzuki A. (2019). Sex Differences in Nonalcoholic Fatty Liver Disease: State of the Art and Identification of Research Gaps. Hepatology.

[44] Kojima S.-I., Watanabe N., Numata M., Ogawa T., Matsuzaki S. (2003). Increase in the prevalence of fatty liver in Japan over the past 12 years: Analysis of clinical background. J. Gastroenterol..

[45] Milani I., Chinucci M., Leonetti F., Capoccia D. (2025). MASLD: Prevalence, Mechanisms, and Sex-Based Therapies in Postmenopausal Women. Biomedicines.

[46] Balakrishnan M., Patel P., Dunn-Valadez S., Dao C., Khan V., Ali H., El-Serag L., Hernaez R., Sisson A., Thrift A.P. (2021). Women Have a Lower Risk of Nonalcoholic Fatty Liver Disease but a Higher Risk of Progression vs Men: A Systematic Review and Meta-analysis. Clin. Gastroenterol. Hepatol..

[47] Harrison S.A., Taub R., Neff G.W., Lucas K.J., Labriola D., Moussa S.E., Alkhouri N., Bashir M.R. (2023). Resmetirom for nonalcoholic fatty liver disease: A randomized, double-blind, placebo-controlled phase 3 trial. Nat. Med..

[48] Newsome P.N., Buchholtz K., Cusi K., Linder M., Okanoue T., Ratziu V., Sanyal A.J., Sejling A.-S., Harrison S.A. (2021). A Placebo-Controlled Trial of Subcutaneous Semaglutide in Nonalcoholic Steatohepatitis. N. Engl. J. Med..

[49] Hartman M.L., Sanyal A.J., Loomba R., Wilson J.M., Nikooienejad A., Bray R., Karanikas C.A., Duffin K.L., Robins D.A., Haupt A. (2020). Effects of Novel Dual GIP and GLP-1 Receptor Agonist Tirzepatide on Biomarkers of Nonalcoholic Steatohepatitis in Patients with Type 2 Diabetes. Diabetes Care.

